# Vascular Flow Phantom of A Cohort-Based Averaged Abdominal Aortic Aneurysm: Design, Fabrication and Characterization

**DOI:** 10.1007/s10439-025-03717-y

**Published:** 2025-04-01

**Authors:** H. Mirgolbabaee, J. R. Nagel, J. Plomp, A. Ghanbarzadeh-Dagheyan, J. A. Simmering, M. Versluis, M. M. P. J. Reijnen, E. Groot Jebbink

**Affiliations:** 1 Multi-Modality Medical Imaging (M3I) Group, Technical Medical Centre, University of Twente, Enschede, The Netherlands; 2 Physics of Fluids (PoF) Group, Technical Medical Centre, University of Twente, Enschede, The Netherlands; 3 Biomedical Photonic Imaging (BMPI) Group, University of Twente, Enschede, The Netherlands; 4 Department of Surgery, Medisch Spectrum Twente, Enschede, The Netherlands; 5 Department of Surgery, Rijnstate Hospital, Arnhem, The Netherlands

**Keywords:** Abdominal aortic aneurysm, Phantom modeling, Phantom fabrication, Particle image velocimetry, Acoustic characterization, Averaged anatomy

## Abstract

**Purpose:**

Vascular flow phantoms are an invaluable tool for in vitro and in silico studies, but their design and fabrication processes are often not reported. In this study**,** a framework is introduced to design and fabricate 3D printable high-fidelity cohort-based averaged abdominal aortic aneurysm (AAA) phantoms.

**Methods:**

AAA geometries of 50 patients were segmented from preoperative computed tomography angiography scans. The segmented geometries and center lumen lines (CLL) were used in an in-house developed algorithm to average the CLL coordinates and corresponding diameters over the entire cohort. The reconstructed averaged anatomy was 3D printed as a thin-walled flow phantom with Formlabs Flexible 80A resin. The acoustic properties of the resin were characterized and the feasibility of flow field quantification inside the phantom with ultrasound particle imaging velocimetry (echoPIV) was investigated.

**Results:**

Comparison between patient-specific models generated by our method and their corresponding reference segmentations, for ten patients, showed a mean Sørensen–Dice similarity coefficient of 0.916 ± 0.21 and the largest distances (5-10% of the lumen diameter) were found at the aneurysmal sac. The Flexible 80A resin had an average speed of sound of 1785 m/s, attenuation of 7.8 dB/mm and density of 1130 kg/m^3^. Volumetric flow profiles obtained with echoPIV in the suprarenal artery (i.e. phantom inlet) matched the flow sensor data.

**Conclusion:**

The reported framework was used to make an averaged, cohort-based AAA model, which showed a good match with its reference model. A 3D printed, thin-walled phantom was made based on this model and the feasibility of flow field quantification inside the phantom was shown.

**Supplementary Information:**

The online version contains supplementary material available at 10.1007/s10439-025-03717-y.

## Introduction

The controllable and reproducible nature of in vitro blood flow phantom studies make them great candidates for optimizing and validating medical imaging techniques such as ultrasound (US) [1], computed tomography angiography (CTA) [2], and magnetic resonance imaging (MRI) [3]. Blood flow phantoms are also used for quality assurance and calibration purposes in medical imaging by providing a reference standard against which the imaging system's performance could be assessed [3, 4]. Moreover, comparative studies were designed by using flow phantoms to investigate the performance, limitations, and advantages of different imaging modalities or devices under standardized conditions, helping to guide clinical decision-making and technological advancements [1, 5]. Flow phantoms are also used in various training and educational sessions, allowing physicians, technicians, and researchers to practice and refine their skills in a controlled simulation environment [6, 7]. Furthermore, in vitro flow phantoms are also instrumental in validating computational models [8, 9]. Therefore, fabrication of high-fidelity flow phantoms holds significant importance across different research domains, as it serves to enhance the efficacy and facilitate the execution of the conducted studies.

Previous in vitro studies conducted with flow phantoms presented several limitations in their methods regarding phantom characterization, design and fabrication. In most cases, the physical properties of the flow phantoms were not reported, making it ambiguous to assess their biomechanical properties, which strongly depend on the chosen materials and geometries. Taneva et al. [10] mentioned that the anatomy and/or design of flow phantoms can be divided into three categories, i.e. simplified, generalized (averaged), and patient-specific. Each of these methods serves its purpose for in vitro studies, depending on the research question, but each also has significant drawbacks. Simplified models are generally limited by their lack of detailed anatomical features and generalizability of results, while patient-specific models show detailed anatomy, but also lack generalizability [11–13]. Generalized models allow us to gain insight into anatomical variations between patient groups, instead of between individual patients, but typically lack detailed anatomical features. Current averaged flow phantoms are often designed by averaging several landmark features such as diameter, vessel segment length, and vessel angulation from the investigated cohort, which result in mostly symmetrical designs [2, 13]. In this approach, the flow-relevant anatomical features (e.g., curvature, tortuosity) are neglected, leading to generalized flow phantoms that are simplified in terms of flow characteristics. It would be beneficial to also have access to models that strike a balance between anatomical detail and generalizability.

Material selection and fabrication techniques not only represent distinct facets of fabricating flow phantoms, but also exhibit a practical interdependence, which are often directly linked with the research question. In many in vitro studies, researchers outsourced the fabrication of the phantom to external companies [2, 11]. Subsequently, there was little control over the choice of material and fabrication procedures, resulting in limited information on the phantom material properties. On the other hand, other researchers fabricated their own block or thin-walled flow phantoms using molding, dip-coating and 3D printing techniques [5, 13]. Materials such as polyvinyl alcohol (PVA), commercial silicone, and polydimethylsiloxane (PDMS) are widely used to fabricate rigid block as well as thin-walled aortic flow phantoms [2, 5, 11, 13–15] using the lost-core technique. Although silicone-based flow phantoms have a long shelf time (i.e. in the order of years), PVA or hydrogel-based flow phantoms, often selected for their tissue-mimicking properties, typically have a shorter shelf time in the order of weeks or months due to water evaporation or mold formation. Moreover, the main challenge in fabricating complex flow phantoms using the lost-core technique is to make the core model, since it should be removed from the containers in a later stage, requiring elaborate degassing schemes to prevent air bubble entrapment. However, 3D printing offers many advantages, being faster, cheaper, relatively mass-producible, and accurately repeatable (i.e., minimum human interactions) in fabricating flow phantoms.

This study introduces a framework for creating high-fidelity realistic generalized flow phantoms, from a cohort-based averaged model. This framework constitutes steps from creating a geometrical model to a 3D printed phantom, intended for flow measurements. These steps are (1) designing the average model; (2) converting the model into a printable 3D geometry; (3) 3D printing the geometry and preparing the flow phantom; and (4) testing the flow phantom. Using this framework, an averaged flow phantom was designed by averaging the CLL of individual patient within the examined cohort, preserving the vessel angulation, curvature, tortuosity of each patient. The proposed framework is suitable for a wide range of vascular structures, however, to showcase our method, a generalized abdominal aortic aneurysm (AAA) flow phantom was designed from a patient cohort. An AAA is a medical condition caused by the permanent dilatation of the abdominal aorta [16]. Depending on the size of the aneurysm and its growth rate, treatment varies from watchful waiting to surgery [17]. With the development of new and advanced imaging and treatment techniques for AAAs, physical and digital vascular phantoms have become an important tool for validation and training purposes.

AAA flow phantoms – and vascular flow phantoms in general – are an invaluable tool for vascular research, optimization and validation of imaging modalities, and training and education. However, studies that use flow phantoms generally do not elaborate on the design and fabrication of their phantoms. In this study we report our framework for design and fabrication of AAA flow phantoms, from segmentation of the relevant anatomy to flow field imaging in 3D printed, thin-walled phantoms.

## Materials & Methods

### Design of the Model

#### Data Selection

The average model was based on segmentations of the aortoiliac region from preoperative CTAs of patients that underwent endovascular aneurysm repair (EVAR). A retrospective cohort of 84 patients was used, from a study by Simmering et al. [18]. This study was approved by the institutional review board (K18-43). The medical data was used in accordance with the regulations by the Dutch Act on Medical Scientific Research Involving Human Being (WMO) and General Data Protection Regulation (AVG). Consent for research participation was waived as it was a retrospective study. Scan parameters of the preoperative CTAs are shown in Table 1. Patients with an iliac aneurysm (iliac diameter > 3 cm) and/or thrombus around the aortic bifurcation were excluded. Patients treated for a ruptured AAA or a dissection were also excluded. Finally, CTAs with insufficient contrast and/or insufficient resolution (slice thickness > 3 mm) for accurate segmentation were excluded as well. The preoperative CTAs of the remaining patients were segmented. For some patients, accurate segmentation of the renal arteries was challenging and these were reassessed on a case-by-case basis by two authors (JN,HM). Different categories of challenging segmentation anatomies were identified, of which the main two relate to presence of multiple renal arteries (MRA), which is a common anatomical variation [19]. When either of the MRAs was dominant (n = 6), only that one was segmented. When the MRAs were similar in diameter (n = 3), the patient was partially included; the renal arteries were excluded, but all other branches were included. The third category were anatomies with a combination of small lumen diameter, orientation parallel to the CTA slices and relatively large slice thickness of 3 mm. These cases (n = 2) were partially included as well.

**Table 1:** Scan parameters from the patient cohort

Scan parameter	Typical values
Tube voltage (kV)	80–100
Tube current (mAs)	75–200
Pixel size (mm)	0.7–0.8
Slice thickness (mm)	2.5–3
CT scanner	Siemens Somatom definition AS

#### Segmentation

**Fig. 1:**
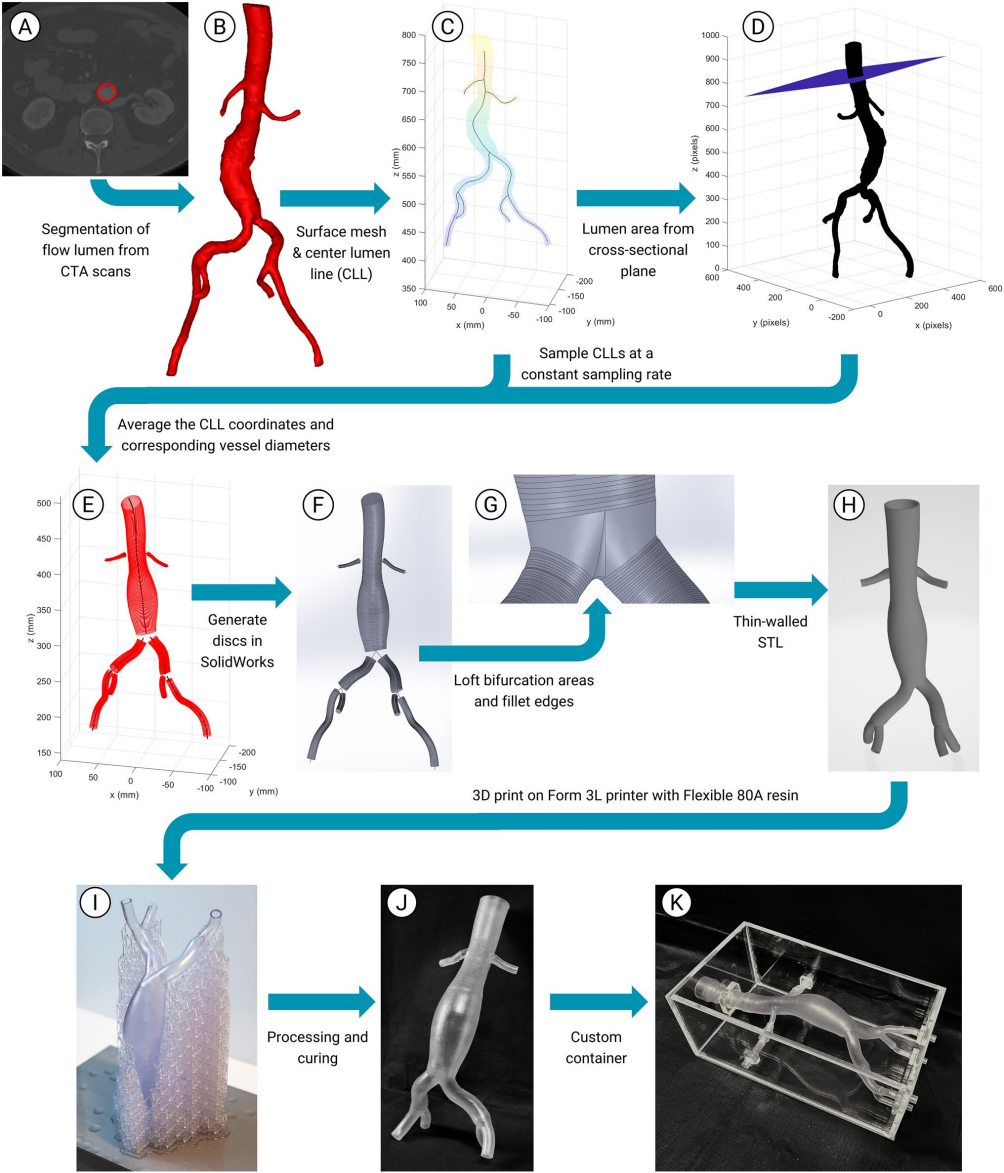
An overview of the phantom design and fabrication process, from CTA scan to final phantom: **a** CTA of abdominal aorta, **b** STL of segmentation, **c** mesh and center lumen line (CLL), **d** cross-sectional slices in volume, to identify and determine the lumen area, **e** averaged CLL and averaged diameters, **f** 3D discs based on CLL and diameter, **g** lofting the bifurcation areas and filleting edges, **h** thin-walled 3D model, **i** phantom with supports, printed vertically to minimize internal supports, **j** printed AAA phantom, **k** phantom positioned in custom-made container

A segmentation of the flow lumen was made for each patient using Mimics software (version 24.0, Materialise, Leuven, Belgium) (Fig. 1a and 1b). For standardization purposes, a predefined segmentation protocol was used and the data was divided between two observers (JN, HM), and segmented according to the protocol. After thresholding and masking the different vessel segments and background, wrapping and smoothing were applied with a maximum of half the slice thickness. The quality of the segmentations was assessed by visual inspection of the contours of the segmentation in the CTA and the segmentation was manually corrected if needed. Both observers checked each other’s final segmentations. Center lumen lines (CLL) were automatically fitted in Mimics with a smoothing factor of 0.5 and were visually inspected and corrected when necessary. The segmentation meshes and CLL coordinates were exported.

#### AAA Averaging Method

The segmentation meshes and the CLL coordinates (Fig. 1c) were imported into Matlab (version R2020a, MathWorks, Natick, MA, USA). All CLLs were resampled to the same number of sample points, equally distanced over the CLL, to normalize differences in anatomy length. Based on their typical length the number of points were 200 for the aorta, 100 for the common iliac arteries, 150 for the external iliac arteries and 75 for the internal iliac arteries and renal arteries. The segmentation meshes were converted to binary volumes and rigidly aligned in the same coordinate system. At each resampled CLL point a cross-sectional plane was defined, perpendicular to the tangent vector of the CLL (Fig. 1d). In this binary cross-sectional plane all boundaries, i.e. edges of lumen cross-sections, were identified. The area inside the boundary was determined. From the cross-sectional lumen area, the diameter of a circular equivalent with the same area was determined. Finally, for each of the resampled CLL points, the coordinates and corresponding diameters were averaged between all patients (Fig. 1e).

#### Generating 3D Model

To generate a 3D model from the averaged data, SolidWorks (version 2023 SP2.1, Dassault Systèmes SOLIDWORKS Corp, Waltham, MA, USA) was used. The average CLL coordinates and the corresponding diameters were imported into SolidWorks. At each CLL point, a ring with the average diameter was defined, and discs were generated between these rings (Fig. 1f). Because no accurate diameters could be determined at the bifurcation areas, these areas were generated separately. Automatic lofting was performed from the most distal disc of the aorta to the most proximal disc of both common iliac arteries (Fig. 1g). The same was done at both iliac bifurcations. The renal arteries were connected to the aorta by extending its most proximal diameter over the part of the CLL where no diameter measurements were available. To avoid sharp edges, filleting was applied to the connections (Fig. 1f). The SolidWorks model was globally smoothed with a smoothing factor of 0.5 in 3-Matic software (Materialise, Leuven, Belgium) and made thin-walled in MeshMixer (version 3.5.474, AutoDesk, San Fransisco, CA, USA) (Fig. 1h).

#### Model Quality

The spread in patient anatomies was evaluated by the standard deviation (SD) of the lumen diameters and the CLL coordinates. For the coordinates, all models were registered to the aortic bifurcation point, and the SD of the CLL coordinates was assessed. Furthermore, the SD of the diameters at each CLL coordinate was assessed.

To test the accuracy of our averaging and model generation method, the method was applied to the meshes and CLLs of ten patients. These ten patients were randomly chosen from the included patients. For each of the patients, the resulting patient-specific model—i.e. the ‘average’ model of just one patient—should then match the original segmentation. The generated meshes and original meshes were compared and the similarity between the two was quantified using the Sørensen–Dice similarity coefficient (SDSC) [20, 21]:

$$SDSC\left(X,Y\right)=\frac{2\left|X\cap Y\right|}{\left|X\right|+\left|Y\right|}$$

Here |X| and |Y| are the number of elements in the full lumen of the two models and |X ∩ Y| is the number of overlapping elements between the two lumens. An SDSC of 1 means that the models are identical, while an SDSC of 0 means that the models don’t have any elements in common. The mean SDSC of ten patients was calculated, together with its standard deviation. Furthermore, for all ten patients, the differences between the original segmentation and patient-specific model made with the averaging script were assessed by calculating and visualizing the distance between the two meshes at each mesh point. The two meshes were registered by manually marking several anatomical landmarks and translating the meshes so that these markers overlapped. At each point of the generated mesh, the distance to the closest point on the segmentation mesh was calculated in MeshLab (version 2022.02, open-source software [22]). These distances were converted to a color index, which was used to visualize the distances between the two meshes.

### Phantom Fabrication

#### 3D printing Thin-Walled Phantom

The final model was 3D printed with a Form 3L (Formlabs, Somerville, MA, USA), using Flexible 80A resin with a wall thickness of 1.5 mm, which is in-line with the reported AAA wall thickness (i.e., median value 1.58 with the SD of 0.64) obtained from different studies [23]. The model was placed upside down, so that the aorta was mostly vertical and the amount of supports at the anterior side of the model was minimized (Fig. 1i). In addition, printing the phantom vertically allows for minimization of internal supports. This was done to minimize the number of support attachment sites that remain on the phantom after processing, which could influence the surface quality of the model. Although with this phantom orientation, the aneurysm sac in the model could be printed free of supports, in the common iliac arteries some internal supports were needed. For these, a support density of 0.5 and touchpoint size 0.6 mm were selected in the PreForm software (Formlabs, Somerville, MA, USA). The internal supports were removed with laparoscopic scissors. Next, the model (Fig. 1j) was flushed and washed with isopropanol alcohol (IPA). Finally, it was left to cure in a closed, transparent container in the lab for two days.

#### Phantom Container

Based on the anatomy of the phantom, a cuboid container and custom-made connectors (Fig. 1k) were designed in SolidWorks, with which arterial endpoints could be aligned. The custom-made connectors were 3D printed using Clear V4 resin (Formlabs, Somerville, MA, USA). The container was fabricated by laser cutting and gluing (Acrifix glue, Röhm GmbH & Co. KG, Darmstadt, Germany) 8-mm Perspex sheets. Laser cutting allows for proper alignment between the branch endpoints and the installed 3D printed connectors, preserving the model anatomy.

### Acoustic Characterization

Although the mechanical properties of Flexible 80A were recently reported [24], to our knowledge, its ultrasound properties are not yet reported in current literature. Hence, we characterized the acoustic properties of this resin to assess the suitability of the 3D printed flow phantoms for ultrasound-based flow imaging modalities. Four Flexible 80A samples were printed with 50-µm resolution directly on the print platform of a Form3 + 3D printer (Formlabs, Somerville, MA, USA); two with a thickness of 1.0 mm and two with a thickness of 2.0 mm. Samples were washed with isopropanol alcohol. To study the influence of curing on the acoustic properties, two samples were cured in an ultraviolet (UV) oven in accordance with the Formlabs’ recommended settings (60 °C, 10 min), while the other two samples were left to dry at room temperature. To prevent additional curing, the samples were covered to avoid exposure to direct sunlight.

**Fig. 2:**
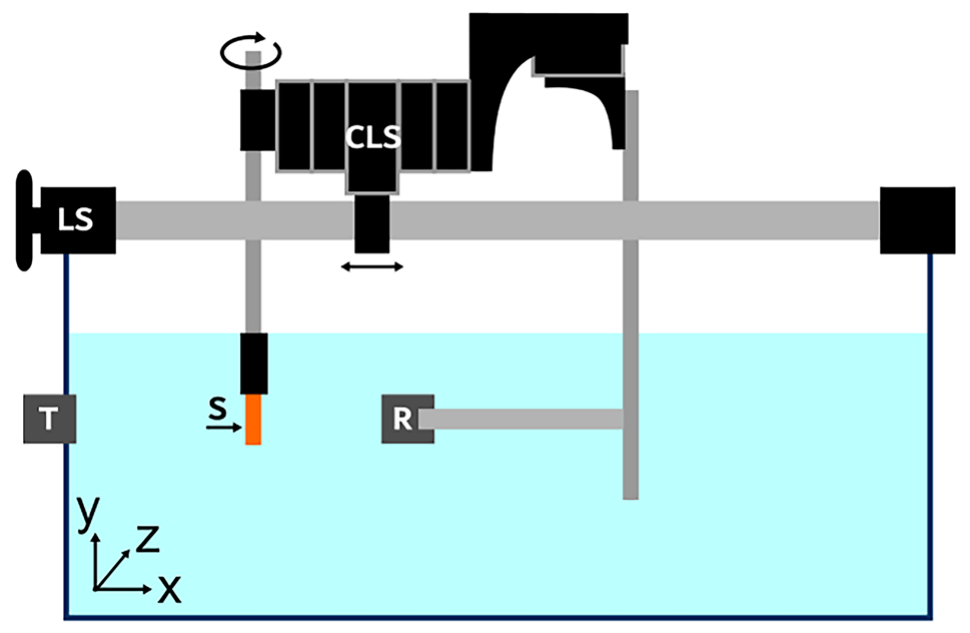
Set-up used for acoustic characterization. A sample (S) is positioned between a transmitting transducer (T) and receiving transducer (R). The x-position of R was first set using the large linear translation stage (LS). A combination of connected linear stages (CLS) was then used for fine alignment of R and S in all directions

The ultrasound setup was built in-house (Fig. 2), consisting of two single-element transducers (V307-SU, Olympus, PA, USA) for transmitting and receiving respectively, a manual translation stage, a sample holder, a tank of degassed water in which all components were submerged, and a control setup to generate and receive the signals. The control setup is described by De Matos et al. [25]. During the measurement, the average water temperature was measured to be 21.8 ± 2 °C, which corresponds to a speed of sound (SoS) of 1488 ± 6 m/s for water [26]. A pulse with a 30-cycle waveform and pressure amplitude of 650 kPa were transmitted with frequencies between 3 and 8.6 MHz at a fixed step size of 0.02 MHz and 10 repetitions at each frequency. Signals of multiple repetitions were averaged before further processing to reduce noise. In addition to the sample measurements, a reference measurement in water was taken.

Attenuation was calculated from the differences in signal amplitude between the 1 mm and 2 mm samples. This eliminated the attenuation due to reflections on the front and back surface of the sample, which is considered similar for both samples. The SoS was calculated from the delay of the signal through the 2 mm sample compared to the reference, which was determined using cross-correlation. The actual dimensions of the samples (as printed) were measured with a digital caliper, with an uncertainty of 0.05 mm. Sample weight was determined using an analytical balance (SECURA224-1S, Sartorius Weighing Technology, Germany), with an uncertainty of 0.1 mg. The measurement uncertainties were used to estimate the uncertainties in the calculated SoS (see Supplementary Information 1).

### Flow Setup, Imaging and PIV Analysis

The fabricated phantom was connected to our in-house in vitro recirculatory AAA flow setup, previously described by Mirgolbabaee et al. [27] Ultrasound contrast microbubbles (SonoVue; Bracco, Milan, Italy) were injected into the recirculatory system. Next, ultrasound particle image velocimetry (echoPIV) measurements were performed using high frame rate (HFR) contrast-enhanced plane wave ultrasound (CEUS) programmed with a Vantage 256 research US system using L12-3 V transducer (Verasonics, Kirkland, WA, USA) to capture flow inside the phantom. To prove the feasibility and evaluate the performance of echoPIV in the 3D printed phantom, we initially performed echoPIV to quantify the flow field in the suprarenal artery (i.e., the inlet of the AAA phantom), which was used to compare with the inlet flow sensor data as ground truth. Thereafter, we chose to image one of the common iliac arteries to present complex flow structures over the course of a cardiac cycle. Details of data acquisition and echoPIV analysis are mentioned in a previous study [27].

## Results

### Average Anatomy Model

#### Model Design

**Fig. 3:**
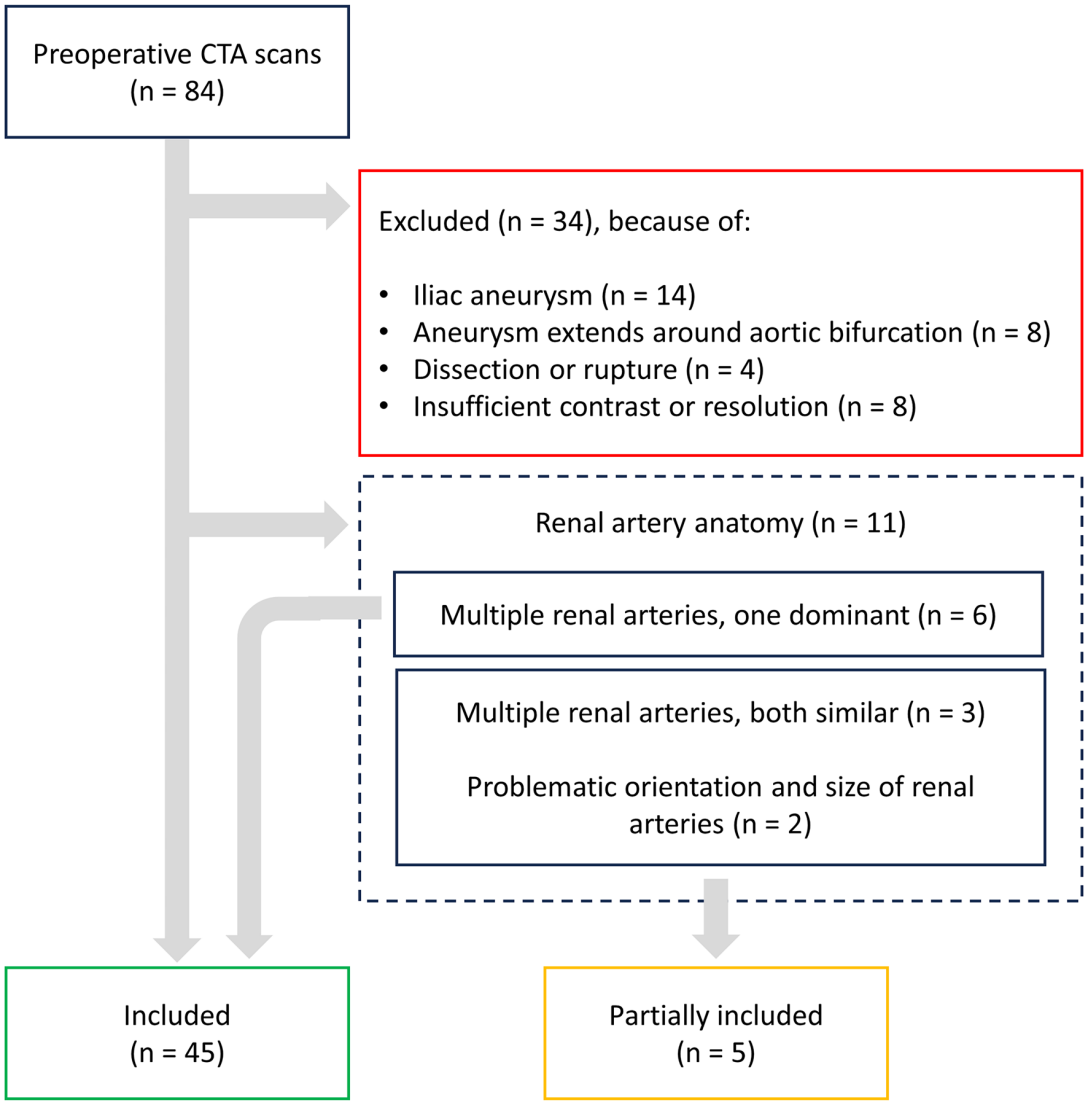
Exclusion criteria for segmentation of preoperative CTA scans from patients that underwent elective EVAR

The abdominal aorta and relevant side branches were segmented for 50 patients, while 35 patients were excluded (Fig. 3). Of the 50 segmentations, 5 were included partially due to the aforementioned challenging segmentation of the renal arteries.

**Fig. 4:**
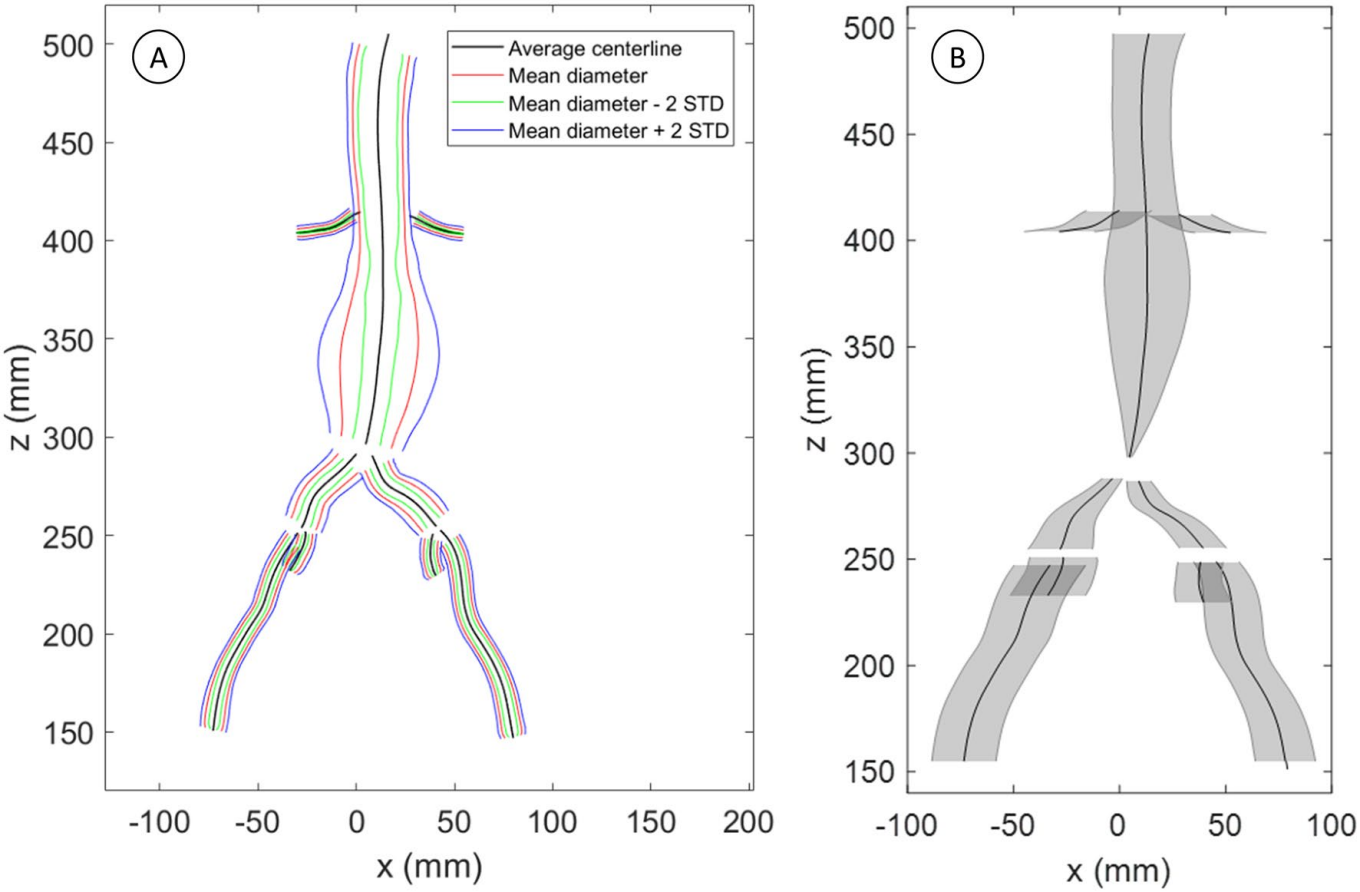
**a** Standard deviation of the diameters between patients; **b** Standard deviation in the horizontal direction of the averaged center lumen line coordinates, with all anatomies registered to the aortic bifurcation

The average vessel diameters and their SD are visualized in Fig. 4a, where the maximum SD is found in the aneurysm sac, with a value of 11.2 mm. The average CLL coordinates and their SD in the horizontal direction are visualized in Fig. 4b. The largest SDs, in three dimensions, of the CLL coordinates for each vessel segment are reported in Table 2 and ranging between 22.0 and 38.7 mm.

**Table 2:** Largest standard deviation, in three dimensions, of the averaged center lumen line coordinates for each section

Vessel section	Maximum SD (mm)
Aorta (including AAA sac)	38.7
Left common iliac artery	22.0
Right common iliac artery	24.3
Left external iliac artery	32.4
Right external iliac artery	32.8
Left internal iliac artery	23.1
Right internal iliac artery	26.0
Left renal artery	29.1
Right renal artery	26.6

#### Model Quality

**Fig. 5:**
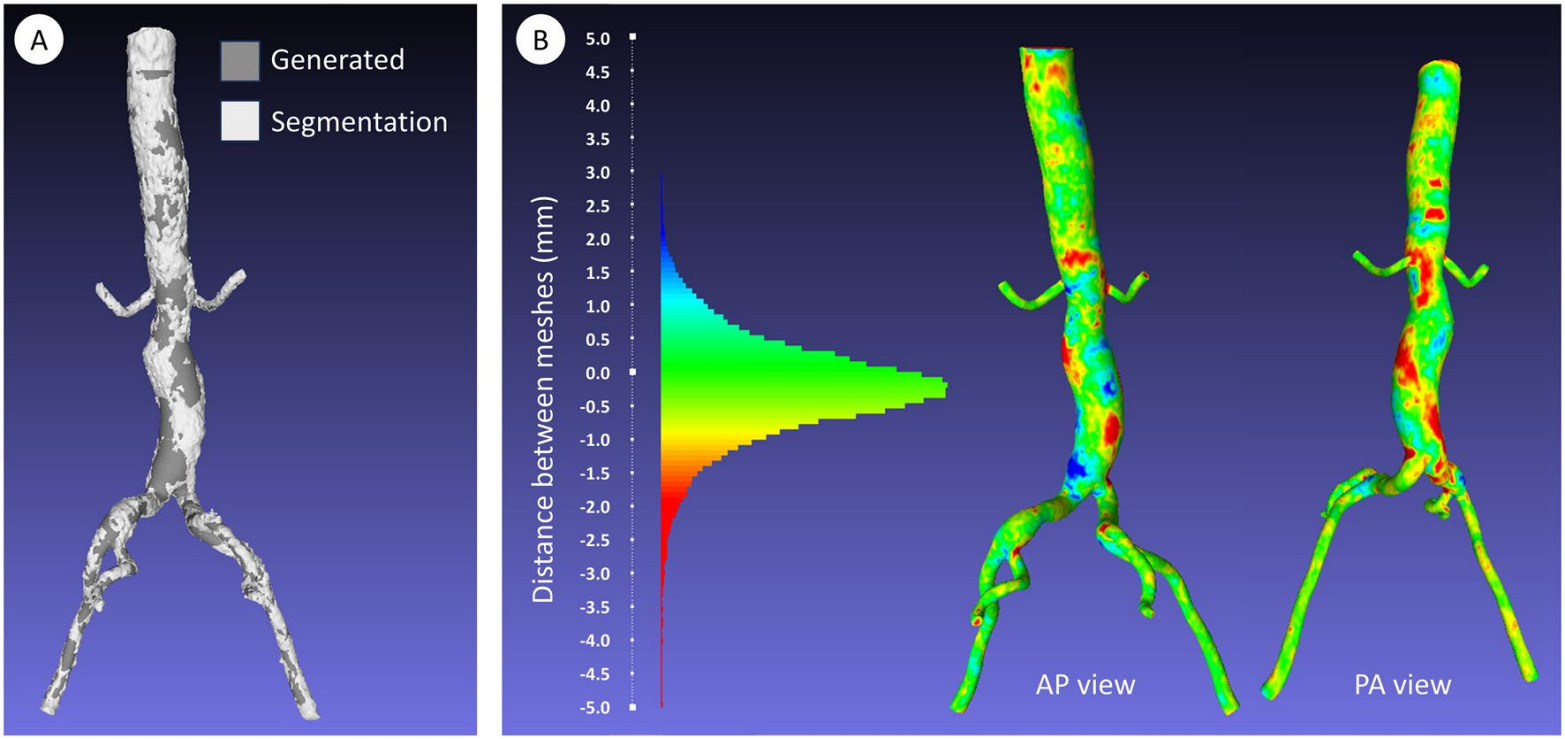
Patient-specific model generated with our method compared to the original segmentation of that patient; **a** segmentation mesh and generated mesh registered on top of each other (dark gray = generated mesh, light gray = segmentation mesh); **b** Visualization of the distance between the meshes in anterior-posterior (AP) view and posterior-anterior (PA) view

The mean Sørensen–Dice similarity coefficient for the meshes of the 10 patients was 0.916 ± 0.021. The generated mesh and original segmentation mesh of one of the patients are shown in Fig. 5a and the visualization of the distance between the meshes is shown in Fig. 5b. The figures for all ten patients are shown in Supplementary Information 1. The largest distances are found in the aneurysm sac, with distances of 1.5 – 3 mm corresponding to a deviation of ~ 5–10% of the maximum flow lumen in the aneurysm sac (which was 31.5 mm for the patient shown in the figure).

### Acoustic Characterization

**Fig. 6:**
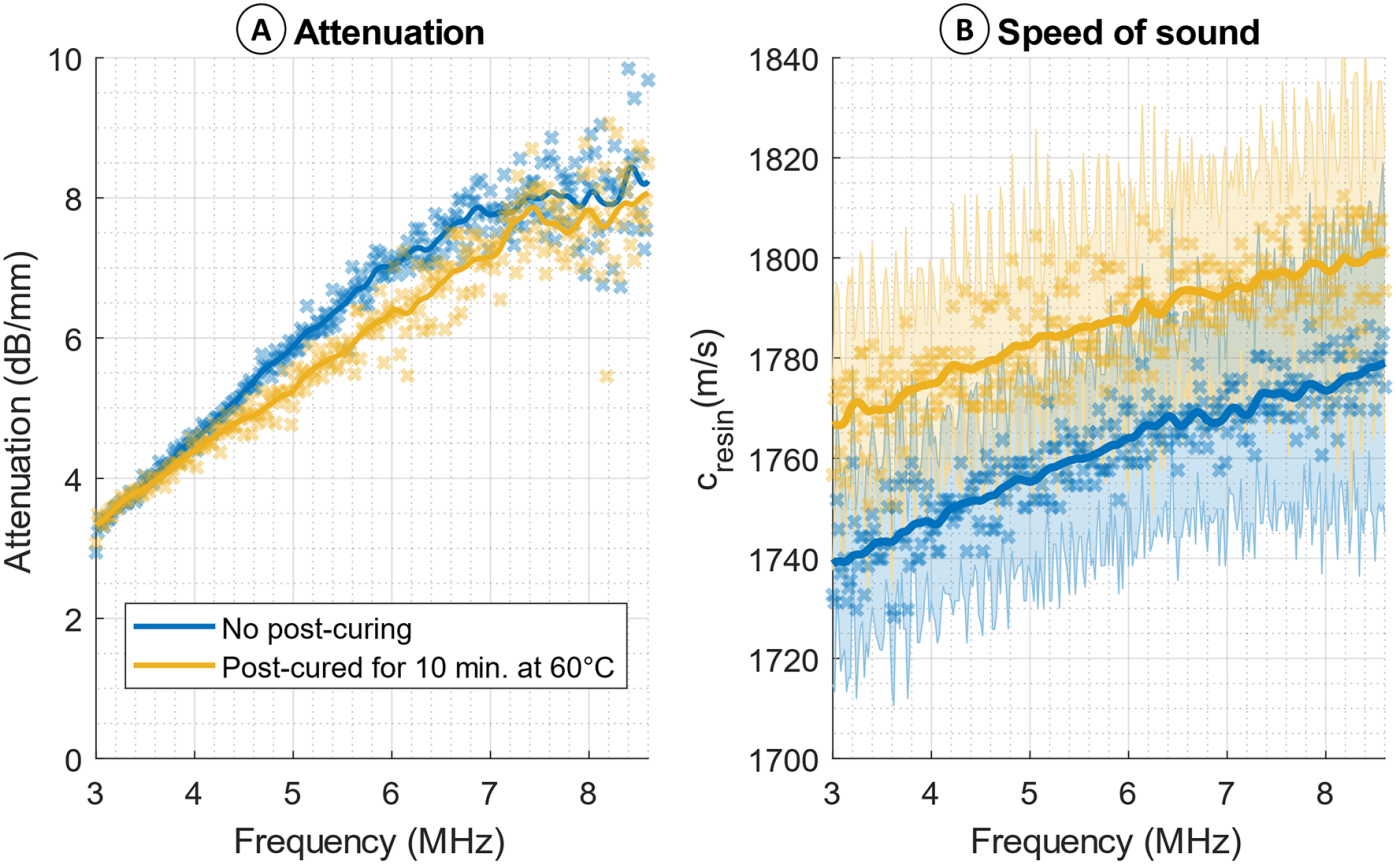
Acoustic characteristics of cured and uncured Flexible 80A resin samples; **a** Attenuation, based on the ratio between a 2 mm and 1 mm thick sample; **b** Speed of sound in cured and uncured resin (based on the 2 mm thick sample). The markers indicate individual measurement points, solid lines represent a gaussian weighted moving average with window size of 16 datapoints (Δf = 0.32 MHz). The shaded error bars represent the uncertainty in the speed of sound calculation

For both the cured and uncured samples (thickness 2.06 ± 0.05 mm and 2.07 ± 0.05 mm, respectively), the density was 1130 ± 30 kg/m^3^. The attenuation and SoS in the samples are presented in Fig. 6, indicating an increasing trend with frequency. At the center frequency (7.5 MHz) of the L12-3v transducer, which was used in the echoPIV measurements, the SoS of the cured and uncured samples was 1800 m/s and 1770 m/s, respectively. Attenuation was 7.67 dB/mm and 8.01 dB/mm, respectively.

### EchoPIV

**Fig. 7:**
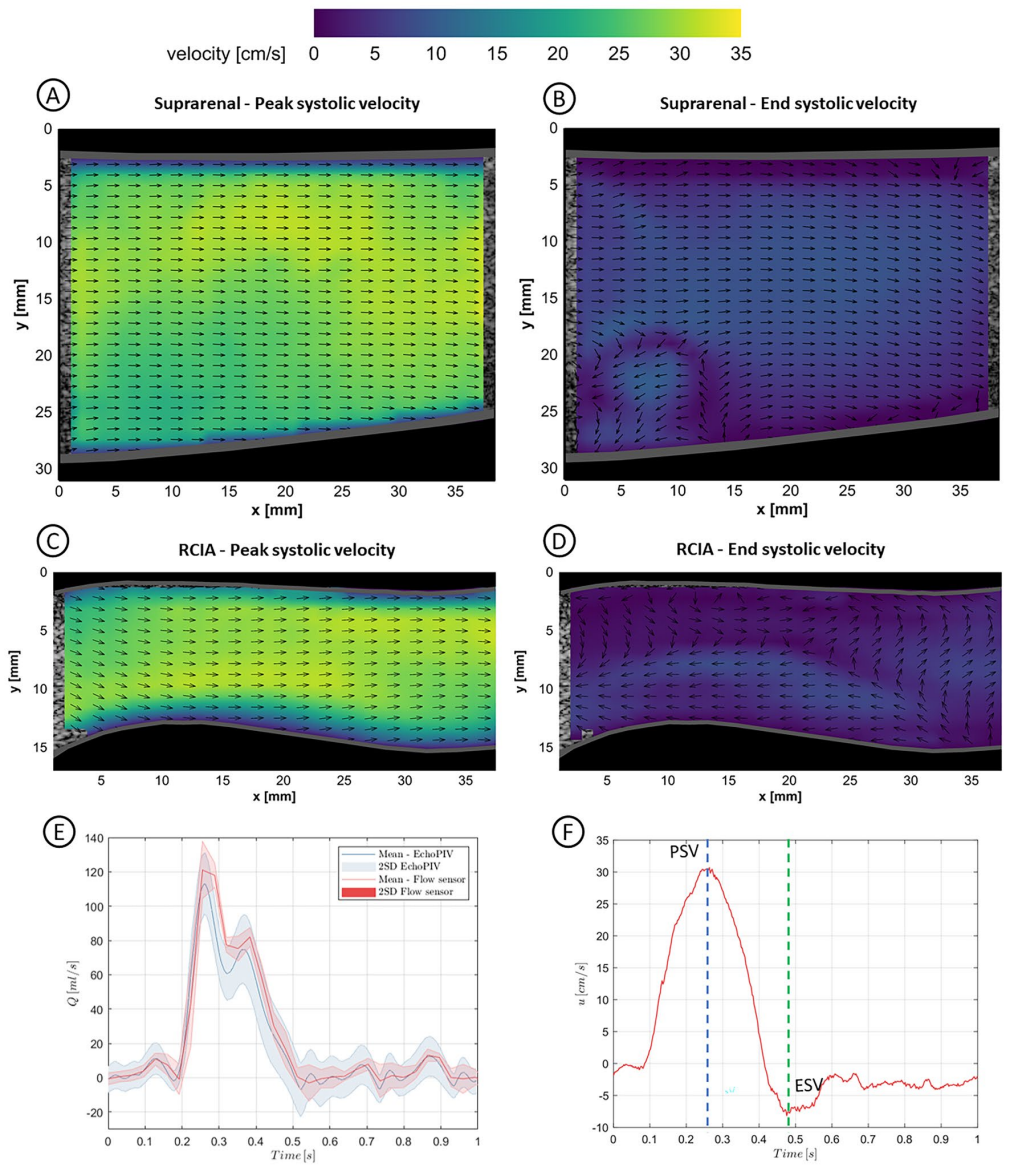
Flow fields in the suprarenal (**a**, **b**) and right iliac (**c**, **d**) arteries at peak systolic velocity and end-systolic velocity time points. Colormap represents the fluid velocity, while the vectors indicate velocity direction (Videos 1 and 2 in SI 2); **e** Volumetric flow profile acquired from the flow sensor compared to the echoPIV driven volumetric flow profile from suprarenal (i.e., inlet) artery; **f** The temporal velocity profile obtained from the center point of the right iliac artery flow lumen. The peak systolic velocity and end-systolic velocity time points are also displayed

The flow fields in the suprarenal artery (i.e., AAA phantom inlet), and right common iliac artery (RCIA) of the flow phantom at peak systolic velocity (PSV) and end-systolic velocity (ESV) time points are depicted in Fig. 7. The temporal velocity profile obtained from the center point of the RCIA flow lumen is displayed in Fig. 7f, where the PSV and ESV time points are highlighted. The 2D velocity vector fields over a cardiac cycle for both suprarenal and RCIA regions are shown in Supplementary Information 2 and 3, respectively. The volumetric flow profile acquired from the inlet flow sensor (Fig. 7e) was compared to the calculated volumetric flow profile from 2D suprarenal spatial velocity profiles from echoPIV measurement. Here, the flow field was assumed to be radially symmetrical in the inlet tube. The volumetric flow profiles were shown together with twice their SD, with an average root mean square error (RMSE) of 7.6 ml/s.

The vector fields were mostly unidirectional during the systolic phase (~0.15–0.4 s), whereas local vortical structures and complex flow fields were observed during the flow reversal phase and diastolic phase (~0.45–1.0 s), during which the flow switched from forward to backward directions and vice versa. The center luminal velocity magnitude at PSV (i.e., maximum forward flow) and ESV (i.e., maximum backward flow) phases were as high as ~ 35 cm/s and ~ 10 cm/s, respectively.

## Discussion

This study presents a cohort-based averaging method and the fabrication of a high-fidelity flow phantom. Furthermore, the fabrication method presented here allows accurate and reproducible fabrication of high-fidelity flow phantoms. The flow imaging feasibility and flow-derived parameters in the averaged anatomy flow phantom was tested using echoPIV, but this framework can be extended to phantoms for other imaging modalities as well. To the best of the authors knowledge this is the first time a phantom pipeline is presented that allows for such versatile and fast phantom generation, only limited by the clinical information available in the used cohort. Furthermore, in this study, segmentations of CTA scans were used, but the averaging method works with any imaging modality for any vessel structure, since it only requires an STL of the segmentation and the corresponding CLL. However, phantom materials should be further investigated and optimized (if needed) for the other imaging modalities.

Quality assessment through the SDSC and the distance between the meshes showed that a patient-specific model generated with our method, matched well to the original segmentation, confirming the reliability of the method. The largest deviations were found in the aneurysm sac (maximum 5–10% of sac diameter). This can be explained by the fact that our averaging method uses a circular equivalent of the cross-sectional lumen area, while the cross-sectional area of the lumen in the aneurysm sac can be more elliptical or irregularly shaped. Furthermore, averaging inherently smoothens small features such as the surface roughness.

The averaged AAA flow phantom designed and fabricated in this study highlights the common anatomy of the aortoiliac region between 50 segmented patient anatomies. However, in combination with clinical outcomes this also allows for comparison of average anatomies of patient groups, for example grouped on complications after EVAR, or studying anatomical extremes, yet within realistic thresholds, for example by comparing anatomies with the 5% highest iliac angulation versus the average. Furthermore, for the flow phantom in this study only the flow lumen was required, but for other applications, such as clinical assessment of the size and growth of the aneurysm sac after EVAR, thrombus could be included as well.

Despite the advantages, 3D printing as a phantom fabrication technique also has several challenges. Thin-walled phantoms often require internal supports and the resulting support attachment sites may influence the surface roughness, which may negatively impact the efficacy of optical and ultrasound-based imaging modalities and may also locally disturb the flow. Another challenge of internal supports is that vascular phantoms can consist of relatively long vessels, which may complicate support removal if supports are too far into the vessel. Furthermore, when printing flexible phantoms, the supports (both external and internal) are also flexible and can therefore deform due to the surface pressure of the resin tank, which can lead to mismatched print layers and subsequent failed prints. 3D printed phantoms also have a size limitation due to the printer’s build platform. To address these inherent 3D printing challenges, we manually oriented the AAA model in the PreForm software, thereby mitigating the risk of potential overhanging forces (refer to Fig 1H). Furthermore, the positioning of the AAA model in this specific orientation resulted in a reduction of the internal supports, which were then carefully removed.

The mechanical properties of the Flexible 80A resin have been reported by Hosseinzadeh et al. [24], in which the authors also developed a coating solution that can be used to fabricate optically transparent phantoms from Flexible 80A resin. They recently demonstrated that coated thin-wall pipe phantoms, using both compliant 3D printing materials from Formlabs (Flexible 80A and Elastic 50A resins), are suitable for both optical PIV and echoPIV [28]. The reported Young’s modulus of the post-cured Flexible 80A samples (range ~ 4–5 MPa) were within the range of values typically found in the human aorta (combined range for both healthy and aneurysmal human aorta: ~ 3.8–5.9 MPa). On the other hand, ultimate tensile strength and elongation values were higher in the Flexible 80A samples compared to the human aorta (ultimate tensile strength ~ 5.1–6.6 MPa in Flexible 80A vs. ~ 1–2.5 MPa in human aorta and elongation ~ 110–170% in Flexible 80A vs. ~ 25–60% in human aorta). However, when applying their photocurable coating on Flexible 80A samples, all reported mechanical properties were within the range of the human’s healthy and aneurysmal aorta [28]. It should be noted that different post-curing settings (temperature and UV exposure duration) can change the mechanical properties of the (coated) Flexible 80A, which requires further investigations to be able to mimic various healthy and diseased vascular biomechanical properties. To further optimize the curing settings of the 3D printed materials, the radial expansion of the phantom vessel wall due to flow pulsatility could be investigated, as well as the possibility of evaluating vessel wall stiffness and blood pressure monitoring using Moens-Korteweg modeling [29]. Furthermore, while the mechanical properties of the 3D printable materials are now compared to a combined range of values for both healthy and aneurysmal human aorta, future studies should investigate the mechanical properties, along with the curing process used, to determine their similarity to the human aorta by distinguishing between healthy and aneurysmal aortic tissue.

Prior to the echoPIV measurements, acoustic characterization experiments were performed to gain insight on the resin echogenicity. Ideally, phantom materials should be acoustically transparent to gain maximum efficiency from ultrasound techniques, causing little attenuation and minimizing artifacts due to any impedance mismatch. The SoS increases by curing the Flexible 80A resin (thus increasing the acoustic impedance mismatch). This can be explained by the increased stiffness of the sample after curing, noting that the density remains the same. Using a tensile test, Morita et al. also showed that the stiffness of the Flexible 80A resin increases after 10 minutes of standard curing [30]. Curing leads to a decrease in attenuation coefficient for a large range of the frequencies measured. For example, at the center frequency of 7.54 MHz (as used in this study), the attenuation decreases from 8.0 dB/mm before curing to 7.7 dB/mm after curing. Since the signal-to-noise ratio obtained from the 2-mm samples was low at frequencies above 7 MHz, this led to an increase in the variance of the attenuation estimates (see Fig. 7a). An extrapolation of a linear fit through the 3–7 MHz data resulted in attenuation estimates of 8.8 dB/mm before curing and 7.8 dB/mm after curing. Depending on the flow phantom applications and required wall thickness, these attenuation differences may be considered in choosing whether or not to cure the phantom after printing. Notably, by extrapolating the attenuation curves reported by Saijo et al. [31], the obtained average attenuation slope of the Flexible 80A (i.e., 1.03 dB/mm/MHz) is in-line with the reported attenuation slope of media and adventitia layers of the healthy blood vessels, being 1.0 ± 0.2 dB/mm and 1.1 ± 0.4 dB/mm, respectively.

In echoPIV, the image plane thickness is determined by the plane wave elevational width, which was < 5 mm for the used probe [32]. Consequently, the plane wave imaging technique used in echoPIV inherently illuminates a wide plane, leading to a larger degree of averaging of the ensemble of microbubble motions. It should also be noted that the volumetric flow rate obtained from echoPIV data is calculated from a measured 2D velocity field, which is extrapolated to the 3D domain by assuming a homogeneous and uniform local radial velocity profile across the entire inlet section (suprarenal region) of the phantom. Therefore, the combination of the afore-mentioned factors explains the systematically lower averaged volumetric flow rate reported by the 2D echoPIV technique. Notably, the validity and limitations of echoPIV measurements have been investigated in several studies, which compared echoPIV with simulated echoPIV, optical PIV, CFD, and analytical velocity profiles, demonstrating acceptable agreement with ground truth velocity fields [8, 28, 33]. Additionally, mass conservation was ensured during the experiments by monitoring the volumetric flow rates from inlet and outlet flow sensors, confirming that the sum of the outlet volumetric flow rates matched the inlet volumetric flow rate.

An alternative to our proposed averaging method could be non-parametric statistical shape modeling (SSM), which is a promising technique in designing average anatomical models from segmented vascular structures [34, 35]. In this approach a mean anatomical shape is obtained by computing a complex mathematical framework, with which the obtained average model can be deformed towards individual anatomical shapes without necessitating point-to-point correspondence. Non-parametric SSM approach can be computationally expensive based on the cohort size, number of meshes in STL file, and 3D volume of the vascular structures, whereas our method is computationally less expensive. Furthermore, using SSM to obtain complex average vascular anatomies with multiple bifurcations, such as the presented AAA case, may be difficult. However, the availability of off-the-shelf, open-source SSM software (such as Deformetrica [36]) and advances in computational efficiency may help mitigate these drawbacks. It could therefore prove beneficial to combine non-parametric SSM and our averaging technique to further improve the framework reported in this work. For instance, non-parametric SSM could be used to replace manual lofting in SolidWorks by computing a realistic averaged model of the bifurcation zones.

## Limitations

While this study provides a higher-fidelity AAA anatomy than most typically used models, there are limitations. Because this method determines the diameter cross-sectional to the CLL, no accurate diameter calculations were possible in the bifurcation areas, and these areas had to be reworked manually. This introduces some observer-dependency which could lead to less accurate bifurcation areas, especially in complex anatomies where a simple loft does not suffice. While the bifurcation areas in our patient-specific model for quality assessment did not show a significant distance between the generated mesh and the reference (i.e. the segmentation mesh), these areas should be investigated further. To improve this part of the method, the areas could be determined more accurately for complex cross-section such as the bifurcation areas, for example by fitting a circular shape to the area, but this could potentially also increase the number of manual steps required, and thus increase the user-dependency of the model.

Because this method is based on averaged anatomical data, and thus only provides the common structure of the arterial vessels, it is not suitable for highly curved or irregular anatomies. Such outliers in a set of anatomical data are not preserved. For such cases, other methods such as SSM may provide a better solution. Moreover, the included CTA scans had a slice thickness between 2 and 3 mm and a pixel resolution in the order of 0.7–0.8 mm. Using CTA scans with smaller slice thicknesses could improve the initial thresholding mask, and thereby the subsequent segmentation steps. A smaller slice thickness would also improve segmentation of the renal arteries, reducing the need for partial segmentations.

Finally, the presented framework makes use of several commercial software tools, due to their availability and the authors’ familiarity with these tools. While each of these tools has open-source alternatives available, their capabilities for easy implementation of the current work flow need to be investigated (for example, we automated the model generation with a macro, so for easy conversion to an open-source alternative, the open-source CAD software should support the use of macros). The most vendor-dependent part of the work flow is the averaging script in Matlab, which can be converted to a Python script with some adjustments.

## Conclusion

In this study, a framework was reported for making averaged, cohort-based AAA phantoms, from segmenting CTA scans to imaging flow in the 3D printed thin-walled phantom, including acoustic characterization of the used resin. Quality assessment of a model generated by our method showed a good match between the generated model and the reference model. Furthermore, feasibility of accurate flow quantification in the 3D printed, thin-walled phantom was shown using echoPIV.

## Supplementary Information

Below is the link to the electronic supplementary material.

Supplementary file1 (pdf 1046 KB)

Supplementary file1 (MP4 50530 KB)

Supplementary file2 (MP4 35721 KB)
