# Supplementary material for: Vascular Flow Phantom of A Cohort-Based Averaged Abdominal Aortic Aneurysm: Design, Fabrication and Characterization
Source: Ann Biomed Eng. 2025 Apr 1;53(6):1439–52. doi: 10.1007/s10439-025-03717-y (PMC12075298; doi:10.1007/s10439-025-03717-y)
Supplement: Supplementary file 1 — Supplementary file1 (pdf 1046 KB) [file 10439_2025_3717_MOESM1_ESM.pdf]

Supplementary Information

Article title:

Vascular flow phantom of a cohort-based averaged abdominal aortic aneurysm: Design, fabrication and characterization

Authors and affiliations:

H. Mirgolbabaee[1,2,], J.R. Nagel[1,2,*], J. Plomp[1,2], A. Ghanbarzadeh-Dagheyan[3], J.A. Simmering[1,4], M. Versluis[2], M. M. P. J. Reijnen[1,5], E. Groot Jebbink[1,5]*

** H. Mirgolbabaee and J.R. Nagel contributed equally to this work and share first authorship*

[1] Multi-Modality Medical Imaging (M3I) Group, Technical Medical Centre, University of Twente, Enschede, The Netherlands

[2] Physics of Fluids (PoF) Group, Technical Medical Centre, University of Twente, Enschede, The Netherlands

[3] Biomedical Photonic Imaging (BMPI) Group, University of Twente, Enschede, The Netherlands

[4] Department of Surgery, Medisch Spectrum Twente, Enschede, The Netherlands

[5] Department of Surgery, Rijnstate Hospital, Arnhem, The Netherlands

Journal:

Annals of Biomedical Engineering

Supplementary Information 1 – Acoustic Characterisation

Attenuation calculation and justification for neglecting thin sample interference

Amplitudes of the signals (average of 10 repetitions) were obtained from the Fourier domain, after cutting off the reflections in the signal. Attenuation was calculated based on the pressure amplitude transmitted through the 1 mm sample (p_{s1}) and the pressure transmitted through the 2 mm sample (p_{s2}), under the assumption of 1D transmission, and assuming the signal is travelling orthogonal to the sample surface. With these assumptions, one can use the following expression:

$$p_{s1} = p_i \cdot T_{wr} \cdot 10^{\left(-\alpha \cdot \frac{d_1}{20}\right)} \cdot T_{rw}$$

where p_i is the amplitude of the incident pressure, α is the attenuation in dB/mm, d_1 is the sample thickness in mm, T_{wr} is the pressure transmission coefficient from water to resin, and T_{rw} is the pressure transmission coefficient from resin to water. Similarly:

$$p_{s2} = p_i \cdot T_{wr} \cdot 10^{\left(-\alpha \cdot \frac{d_2}{20}\right)} \cdot T_{rw}$$

The ratio of the two signals then becomes:

$$\frac{p_{s1}}{p_{s2}} = \frac{p_i \cdot T_{wr} \cdot 10^{\left(-\alpha \cdot \frac{d_1}{20}\right)} \cdot T_{rw}}{p_i \cdot T_{wr} \cdot 10^{\left(-\alpha \cdot \frac{d_2}{20}\right)} \cdot T_{rw}} = 10^{\frac{-\alpha(d_1 - d_2)}{20}}$$

such that:

$$\alpha = -\frac{20 \log_{10}\left(\frac{p_{s1}}{p_{s2}}\right)}{d_1 - d_2} = \frac{20 \log_{10}\left(\frac{p_{s1}}{p_{s2}}\right)}{d_2 - d_1}$$

This method depends on the assumption that interference can be neglected. In thin samples, depending on the wavelength and sample thickness, a signal can have interference with the internal reflections within the sample. If these are significant with respect to the total signal amplitude, then the attenuation can't be estimated from these for an arbitrary sample thickness.

Fig. S1.1 shows the attenuation for all 4 samples, where the attenuation ratio is calculated as the ratio between the signal amplitude for a water reference (p_{ref}) and the signal transmitted through a sample (p_s). Although at a small scale, some oscillations are observed (not visible in the graph), these were deemed sufficiently small such that the attenuation can be assumed to be dominant over the effect of the possible internal reflections. This motivates the use of the ratio of the signal amplitude transmitted through the 1 mm and 2 mm signals to calculate attenuation.

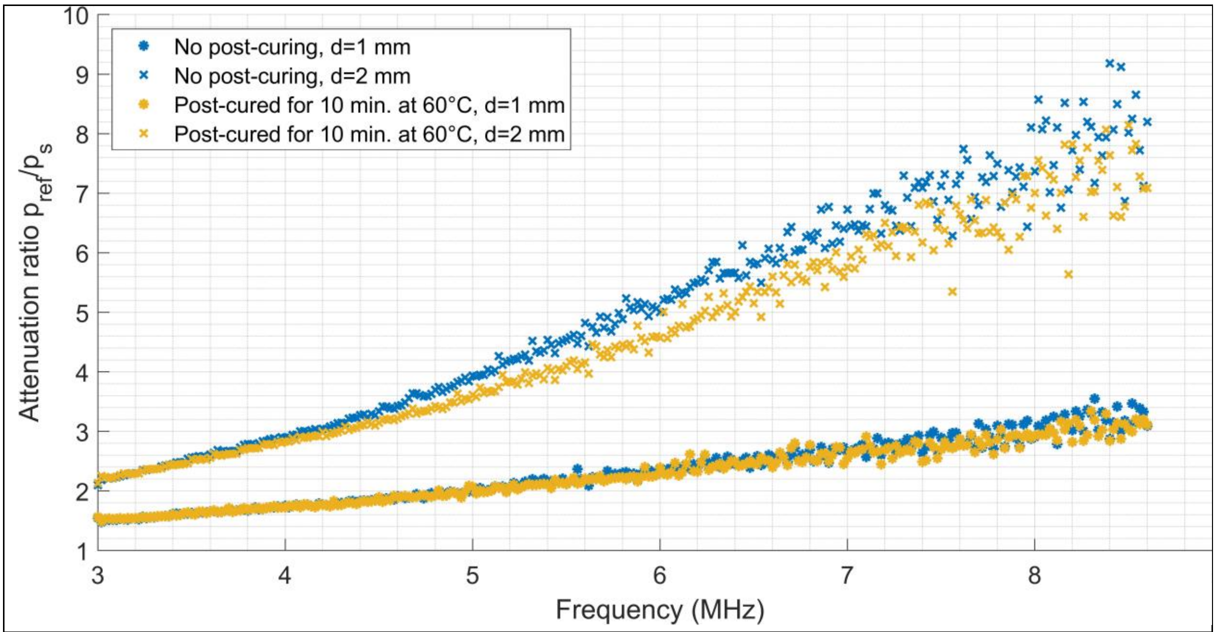

Figure S1.1*: Ratio of the signal amplitude for a water reference p_{ref} and for the signal transmitted through a sample, p_s for all four samples. The oscillations in the individual curves were considered sufficiently minimal to assume the effects of thin-sample interference could be neglected.*

Speed of sound calculation including uncertainty estimation

The following expression was used to calculate speed of sound in the Flexible 80A resin:

$$c_r = \frac{dc_w}{c_w \Delta t + d}$$

Where c_r is the speed of sound, d is the sample thickness, c_w is the speed of sound in water, and Δt is the estimated delay between the arrival time of the signal in the reference measurement and the arrival time when the signal travels through the sample. The uncertainty in the above calculation is defined as:

$$\Delta c_r = \left|\frac{\partial c_r}{\partial d}\right| \Delta \mathrm{d} + \left|\frac{\partial c_r}{\partial c_w}\right| \Delta \mathrm{c_w} + \left|\frac{\partial c_r}{\partial \Delta \mathrm{t}}\right| \Delta\Delta \mathrm{t}$$

Where $\Delta d, \Delta c_w$ and $\Delta\Delta t$ are the measurement uncertainties in the respective quantities. Based on the given definition of c_r, the partial derivatives are:

$$\left|\frac{\partial c_r}{\partial d}\right| = \left|\frac{c_w(\Delta t c_w + d) - dc_w}{(\Delta t c_w + d)^2}\right|$$

$$\left|\frac{\partial c_r}{\partial c_w}\right| = \left|\frac{d(\Delta t c_w + d) - dc_w \Delta t}{(\Delta t c_w + d)^2}\right|$$

$$\left|\frac{\partial c}{\partial \Delta t}\right| = \left|\frac{-dc_w^2}{(\Delta t c_w + d)^2}\right|$$

To calculate Δc_r, the measurement uncertainties are required. As described in the method section, c_w was estimated at 1488±6 m/s, where the uncertainty arises from the uncertainty in the temperature measurement. The uncertainty in the caliper measurements was estimated to be 0.05 mm. The caliper has an uncertainty of 0.01 mm, but due to the flexibility of the sample, some extra uncertainty was introduced. $\Delta\Delta t$ is the uncertainty in the delay, which was estimated as follows:

At each measurement frequency, the signals of the sample and reference measurement were first aligned by shifting one signal by Δt, which was estimated using cross-correlation. Next, the position of the highest 24 peaks was determined in the sample and reference measurement. The largest difference in position (1 ns resolution due to sampling rate) was used as the uncertainty $\Delta\Delta t$.

Supplementary Information 2 – Mesh distance for quality assessment

For 10 randomly chosen patients, the differences between original segmentation and patient-specific model, made with the averaging script, were assessed by calculating and visualizing the distance between the two meshes at each mesh point. The two meshes were registered by manually marking several anatomical landmarks and translating the meshes so that these markers overlapped. At each point of the generated mesh, the distance to the closest point on the segmentation mesh was calculated in MeshLab (version 2022.02, open-source software). These distances were converted to a color index, which was used to visualize the distances between the two meshes. In the manuscript, the results for one patients are shown, to illustrate the method and results. This supplementary information shows the mesh-distance figures for all 10 patients (Fig. S2.1 to S2.10).

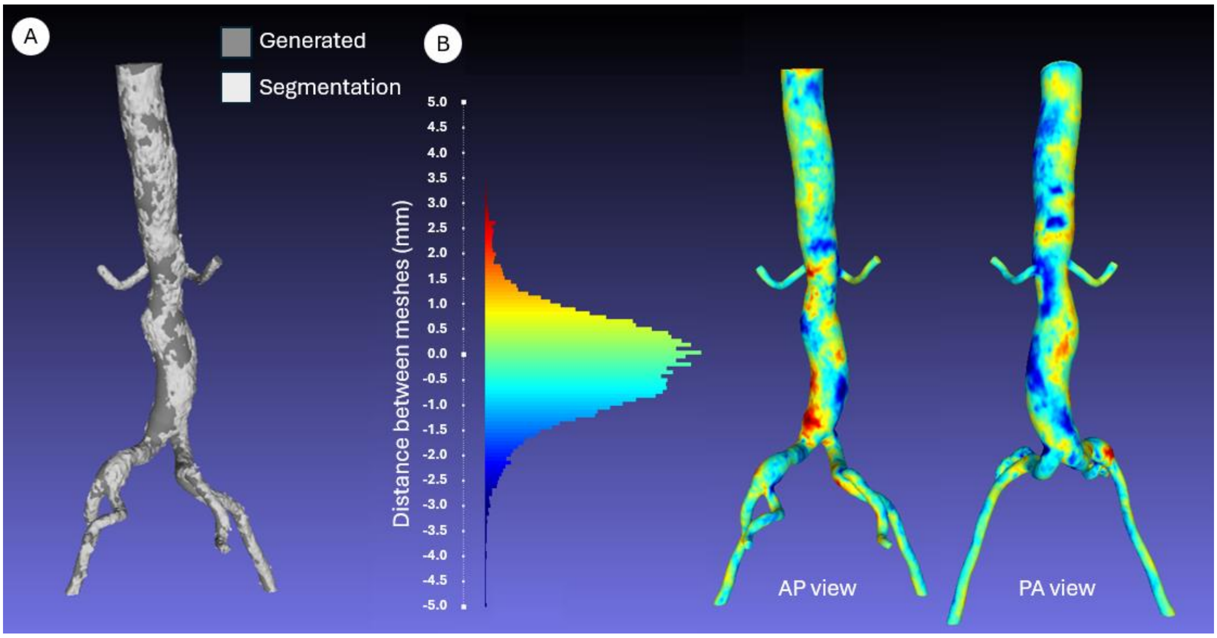

***Fig. S2.1**. Patient-specific model generated with our method compared to the original segmentation of patient 002; a) segmentation mesh and generated mesh registered on top of each other; dark grey = generated mesh, light grey = segmentation mesh, b) Visualization of the distance between the meshes in anterior-posterior (AP) view and posterior-anterior (PA) view*

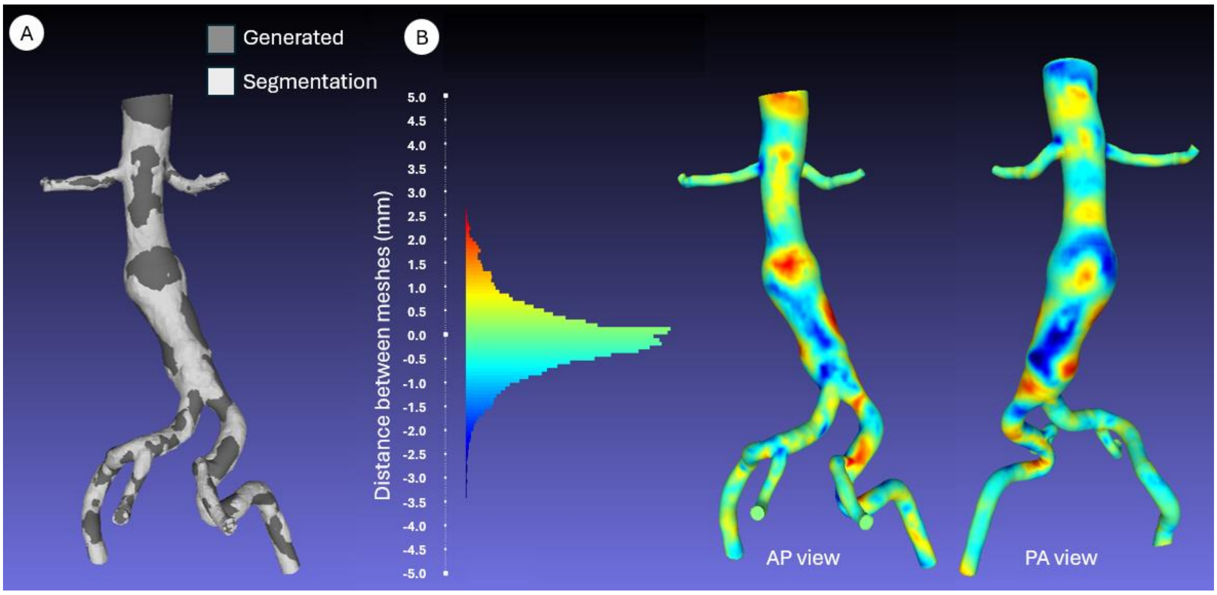

Fig. S2.2. *Patient-specific model generated with our method compared to the original segmentation of patient 008; a) segmentation mesh and generated mesh registered on top of each other; dark grey = generated mesh, light grey = segmentation mesh, b) Visualization of the distance between the meshes in anterior-posterior (AP) view and posterior-anterior (PA) view*

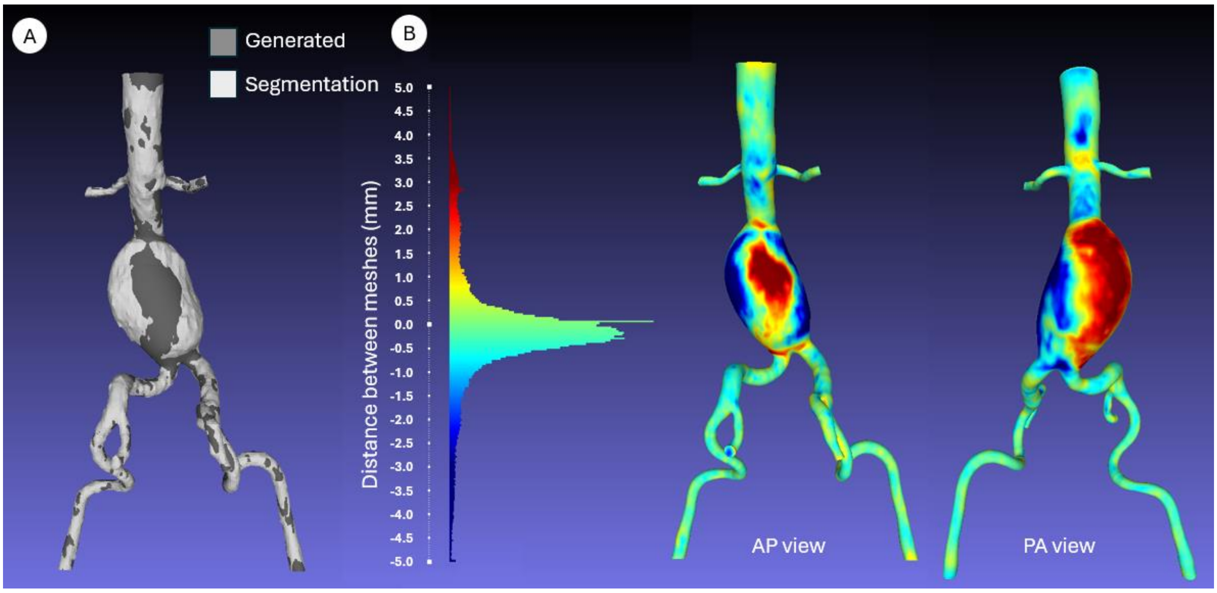

Fig. S2.3. *Patient-specific model generated with our method compared to the original segmentation of patient 009; a) segmentation mesh and generated mesh registered on top of each other; dark grey = generated mesh, light grey = segmentation mesh, b) Visualization of the distance between the meshes in anterior-posterior (AP) view and posterior-anterior (PA) view*

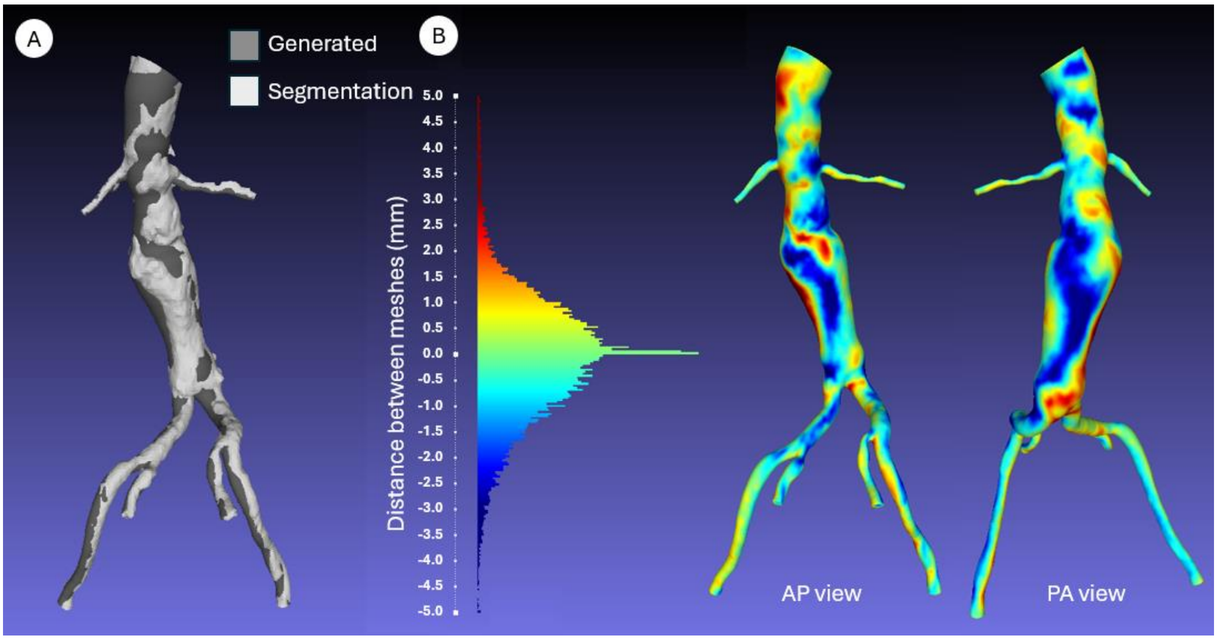

Fig. S2.4. *Patient-specific model generated with our method compared to the original segmentation of patient 015; a) segmentation mesh and generated mesh registered on top of each other; dark grey = generated mesh, light grey = segmentation mesh, b) Visualization of the distance between the meshes in anterior-posterior (AP) view and posterior-anterior (PA) view*

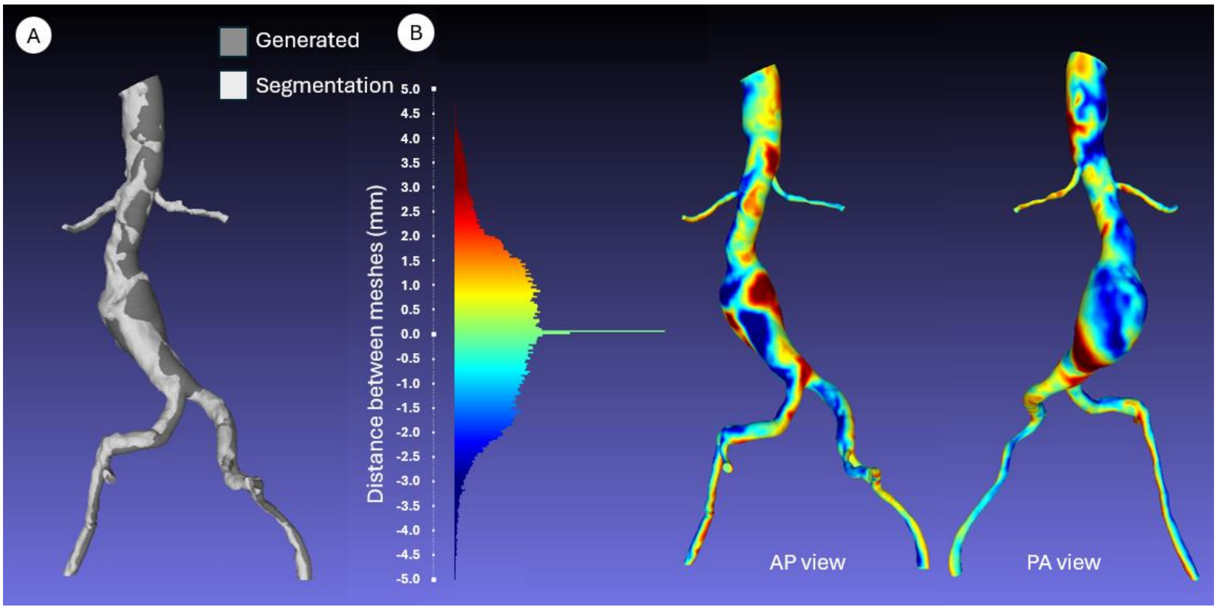

Fig. S2.5. *Patient-specific model generated with our method compared to the original segmentation of patient 024; a) segmentation mesh and generated mesh registered on top of each other; dark grey = generated mesh, light grey = segmentation mesh, b) Visualization of the distance between the meshes in anterior-posterior (AP) view and posterior-anterior (PA) view*

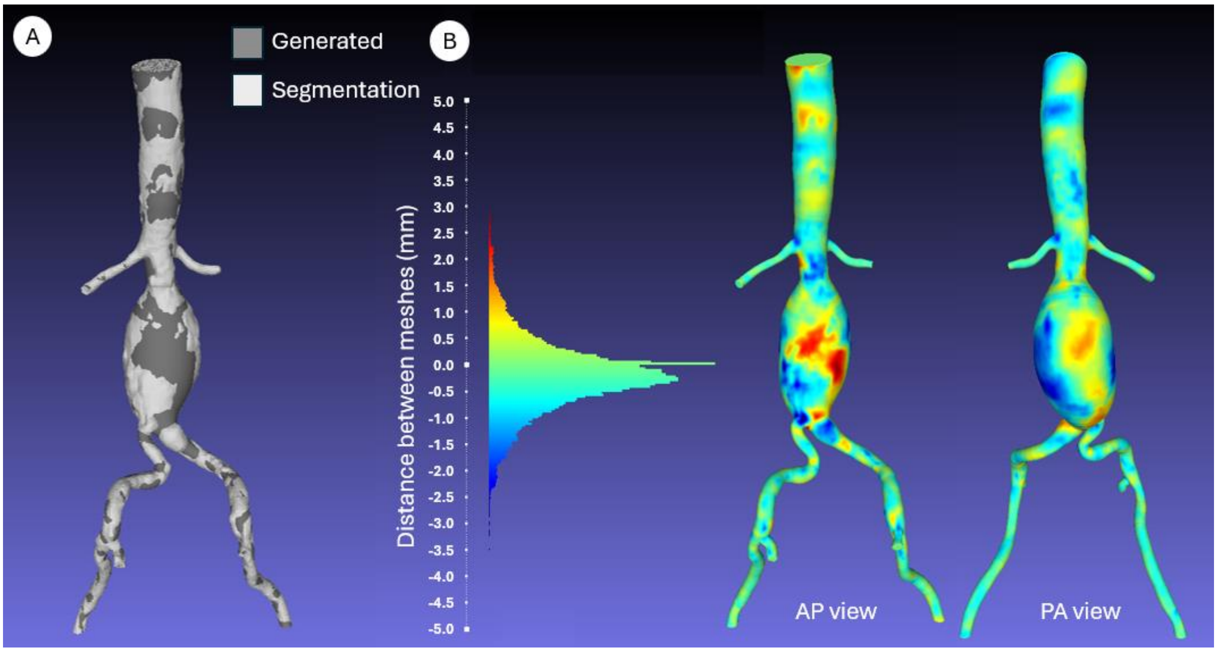

Fig. S2.6. *Patient-specific model generated with our method compared to the original segmentation of patient 033; a) segmentation mesh and generated mesh registered on top of each other; dark grey = generated mesh, light grey = segmentation mesh, b) Visualization of the distance between the meshes in anterior-posterior (AP) view and posterior-anterior (PA) view*

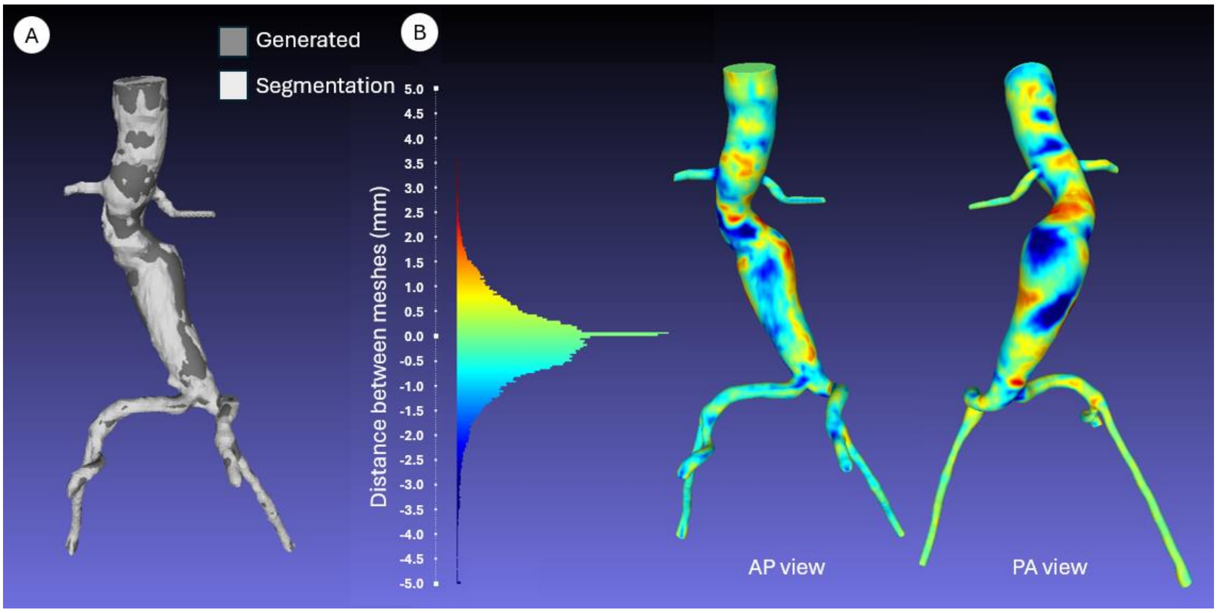

Fig. S2.7. *Patient-specific model generated with our method compared to the original segmentation of patient 039; a) segmentation mesh and generated mesh registered on top of each other; dark grey = generated mesh, light grey = segmentation mesh, b) Visualization of the distance between the meshes in anterior-posterior (AP) view and posterior-anterior (PA) view*

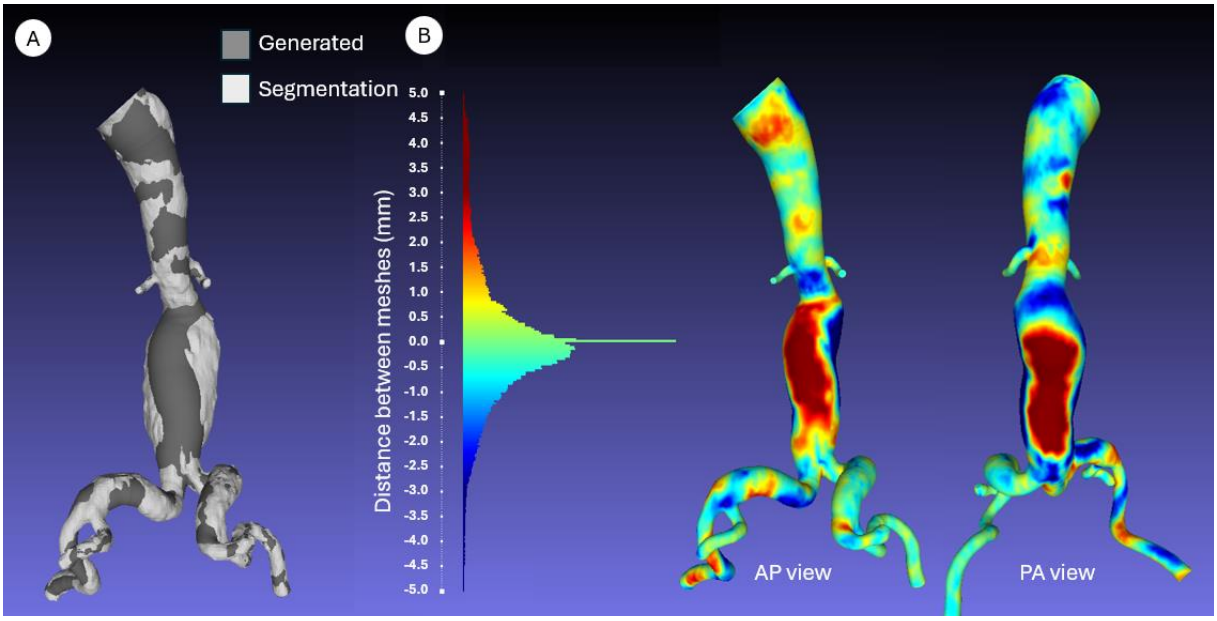

Fig. S2.8. *Patient-specific model generated with our method compared to the original segmentation of patient 059; a) segmentation mesh and generated mesh registered on top of each other; dark grey = generated mesh, light grey = segmentation mesh, b) Visualization of the distance between the meshes in anterior-posterior (AP) view and posterior-anterior (PA) view*

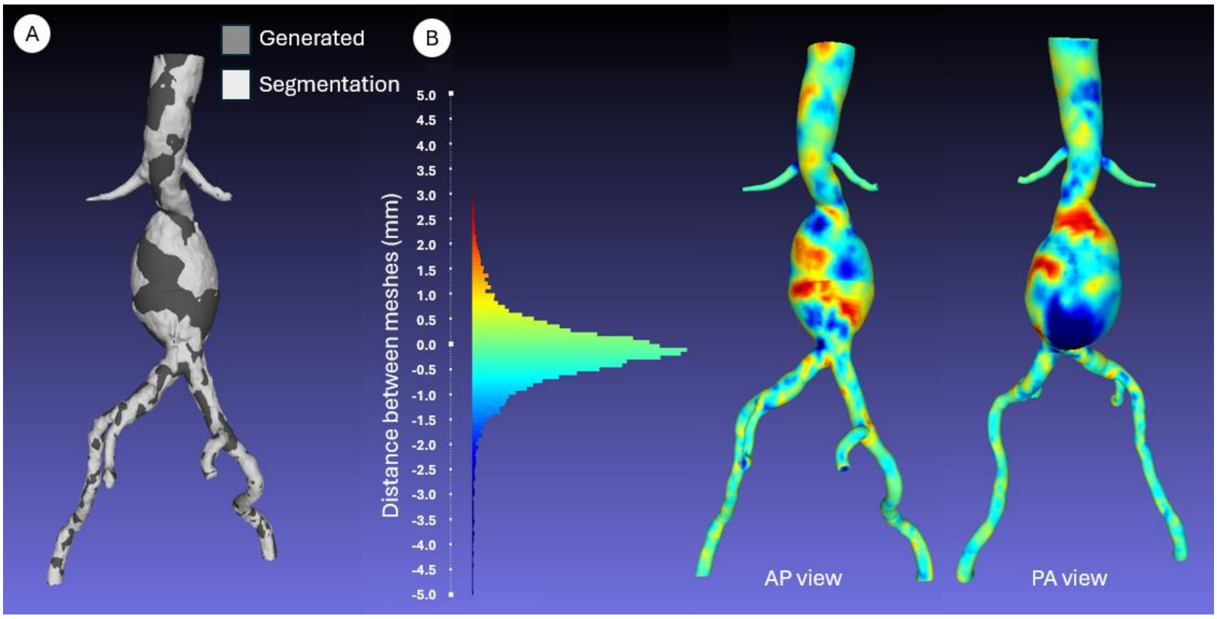

Fig. S2.9. *Patient-specific model generated with our method compared to the original segmentation of patient 077; a) segmentation mesh and generated mesh registered on top of each other; dark grey = generated mesh, light grey = segmentation mesh, b) Visualization of the distance between the meshes in anterior-posterior (AP) view and posterior-anterior (PA) view*

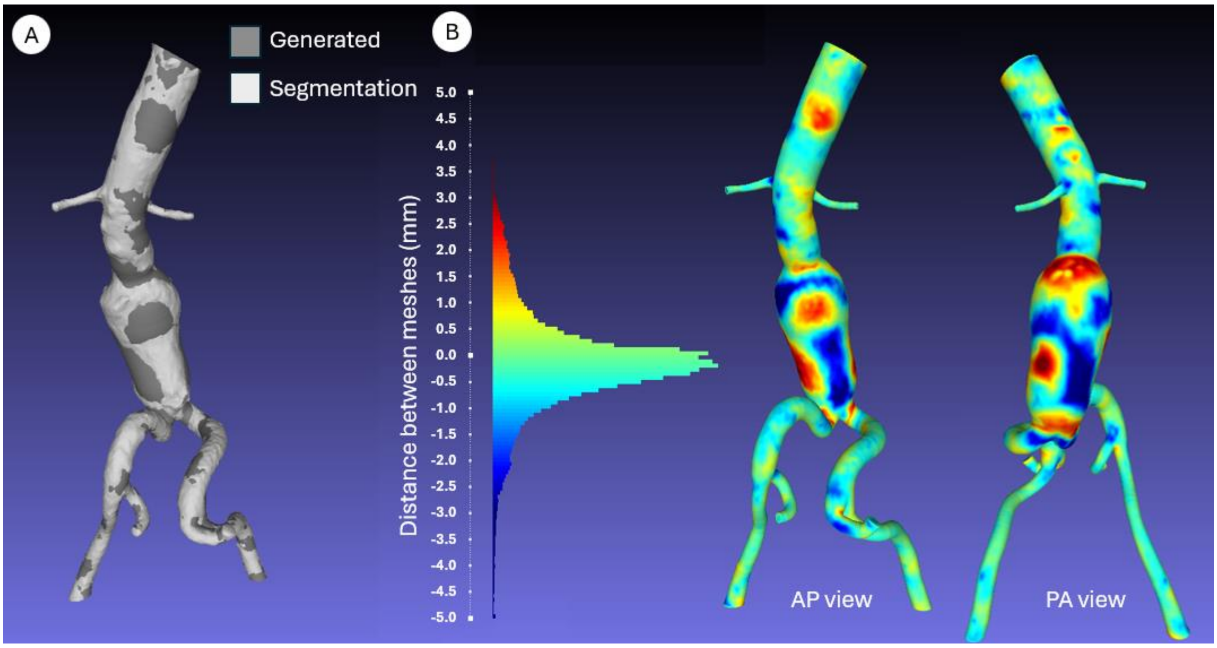

Fig. S2.10. *Patient-specific model generated with our method compared to the original segmentation of patient 084; a) segmentation mesh and generated mesh registered on top of each other; dark grey = generated mesh, light grey = segmentation mesh, b) Visualization of the distance between the meshes in anterior-posterior (AP) view and posterior-anterior (PA) view*

Supplementary Information 3 – echoPIV videos

echoPIV videos are attached as separate files:

- Video 1: Velocity fields obtained from the suprarenal artery (i.e., inlet of the flow phantom).
- Video 2: Velocity fields obtained from the right common iliac artery.
